# A deep learning approach to identifying immunogold particles in electron microscopy images

**DOI:** 10.1038/s41598-021-87015-2

**Published:** 2021-04-08

**Authors:** Diego Jerez, Eleanor Stuart, Kylie Schmitt, Debbie Guerrero-Given, Jason M. Christie, William E. Hahn, Naomi Kamasawa, Michael S. Smirnov

**Affiliations:** 1 Max Planck Florida Institute for Neuroscience, 1 Max Planck Way, Jupiter, FL 33458 USA; 2 Department of Mathematical Sciences, Florida Atlantic University, 777 Glades Rd, Boca Raton, FL 33431 USA

**Keywords:** Scanning electron microscopy, Scientific data, Software

## Abstract

Electron microscopy (EM) enables high-resolution visualization of protein distributions in biological tissues. For detection, gold nanoparticles are typically used as an electron-dense marker for immunohistochemically labeled proteins. Manual annotation of gold particle labels is laborious and time consuming, as gold particle counts can exceed 100,000 across hundreds of image segments to obtain conclusive data sets. To automate this process, we developed Gold Digger, a software tool that uses a modified pix2pix deep learning network capable of detecting and annotating colloidal gold particles in biological EM images obtained from both freeze-fracture replicas and plastic sections prepared with the post-embedding method. Gold Digger performs at near-human-level accuracy, can handle large images, and includes a user-friendly tool with a graphical interface for proof reading outputs by users. Manual error correction also helps for continued re-training of the network to improve annotation accuracy over time. Gold Digger thus enables rapid high-throughput analysis of immunogold-labeled EM data and is freely available to the research community.

## Introduction

Biological samples, including brain tissue, contain many types of proteins such as membrane-integrated ion channels, transporters, receptors, and enzymes, which mediate the interactions of cells to other cells or their environment. Many of these proteins are uniquely distributed in cells, and visualizing their subcellular localization and density helps to provide a better understanding of their function. EM offers the highest resolution for identifying protein distributions. Immunohistochemical-based protein identification in EM is accomplished by attaching an electron dense marker, such as gold nanoparticles, to the protein of interest for visualization during imaging. EM immunolabeling techniques that use colloidal gold particles include both post-embedding approaches as well as the freeze-fracture replica immunogold labeling (FRIL) method. The latter provides for exacting quantification of membrane protein distributions with high specificity and efficacy^1,2^. A significant bottleneck in the analysis workflow of immunogold-labeled EM datasets is identifying and characterizing the location of individual gold particles in images. Conventionally, gold particle identification is accomplished manually by human annotators. For large datasets, image analysis is inherently time consuming, tedious, and prone to error due to the shifting judgement of the annotator over time. Thus, the seemingly simple task of counting gold nanoparticles is actually a tiresome exercise of manual labor, with the drawback of inconsistency, precluding achievement of statistical depth gained with repeat, accurate measurements.

Computer vision technology has been employed in recent years to automate gold particle detection in EM images using a standardized, unwavering set of criteria^3^. However, these approaches require frequent adjustments of individual parameters (e.g., thresholding and/or circularity calculations) to accurately identify gold particles. In addition, computer-vision-based gold particle counting is difficult in FRIL images due to the “shadows” cast by objects, as well as the undulating topography of the fractured sample. These detection inconsistencies persist even when employing advanced adaptive thresholding approaches to differentiate gold particles across an uneven background^4^. Recently, an accurate, semi-automated method was developed to detect gold particles in FRIL samples by using image manipulations in tandem with a classification model^5^. However, this program cannot handle large-sized images and does not support automatic detection of different sized gold particles within a single image. In summary, traditional computational techniques have underwhelmed in their ability to count gold particles in heterogeneous backgrounds common to EM biological samples.

Within the past decade, there has been a rapid shift in the standard approach taken towards computer vision, where tools have began relying on large, multilayered neural networks, also known as deep learning. Deep learning has been used to analyze various kinds of biological data, some examples of which include animal behavior^6^, medical images^7^, and protein structure^8^. To increase the capability, speed, and accuracy of colloidal gold particle detection in EM images, we created Gold Digger, a deep-learning-based software tool capable of autonomous gold particle identification after human training. The algorithm behind Gold Digger is based on pix2pix, a generative adversarial network^9^. By compounding the capability of two convolutional neural networks, gold particle analysis is achieved in a fast and efficient manner and requires a modicum of labeled training data. Gold Digger is capable of identifying gold particles of any size, and then categorizing these particles into different size groups. Gold Digger’s applicability on completely novel image sets, along with its generalizability, is a significant improvement relative to other solutions for gold particle identification in FRIL images.

## Results

### Network design

**Figure 1:**
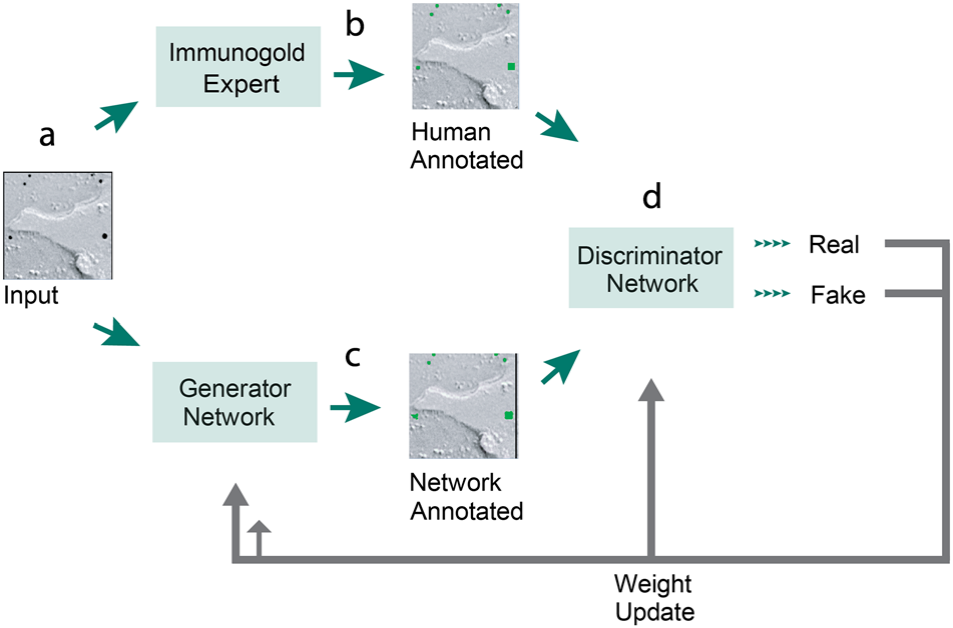
Network Structure of Gold Digger. (**a**) A Cropped FRIL image used for input (**b**) Ground truth image annotated by human experts (**c**) Network-generated image with annotations **d.** Discriminator network to discern between real and fake images.

For automated gold particle detection in EM images, we chose to employ a conditional generative adversarial network (cGAN) architecture (Fig. 1), based on a previously developed approach named Pix2Pix^9^. A cGAN uses variant convolutional neural networks for both generator and discriminator networks. During training, an input image (Fig. 1a) is assigned both human-generated annotations (Fig. 1b), and annotations assigned by the generator network (Fig. 1c). Ground truth annotations are applied as a square pixel mask over each gold particle (Fig. 1b). The annotated images are then fed to the discriminator network, which is tasked with discerning between the outcome image created by the generator (“fake”), and the ground truth created by a human expert (“real”) (Fig. 1d). The weights of both the generator and discriminator networks are then updated to increase overall annotation performance by directing the generator network to improve in annotating the images that the discriminator network is able to detect as false. The generator network’s ability to annotate images improves throughout training: the constant process of weight re-adjustments improves overall network performance, resulting in its ability to generate human-level image annotations^10^.

**Figure 2:**
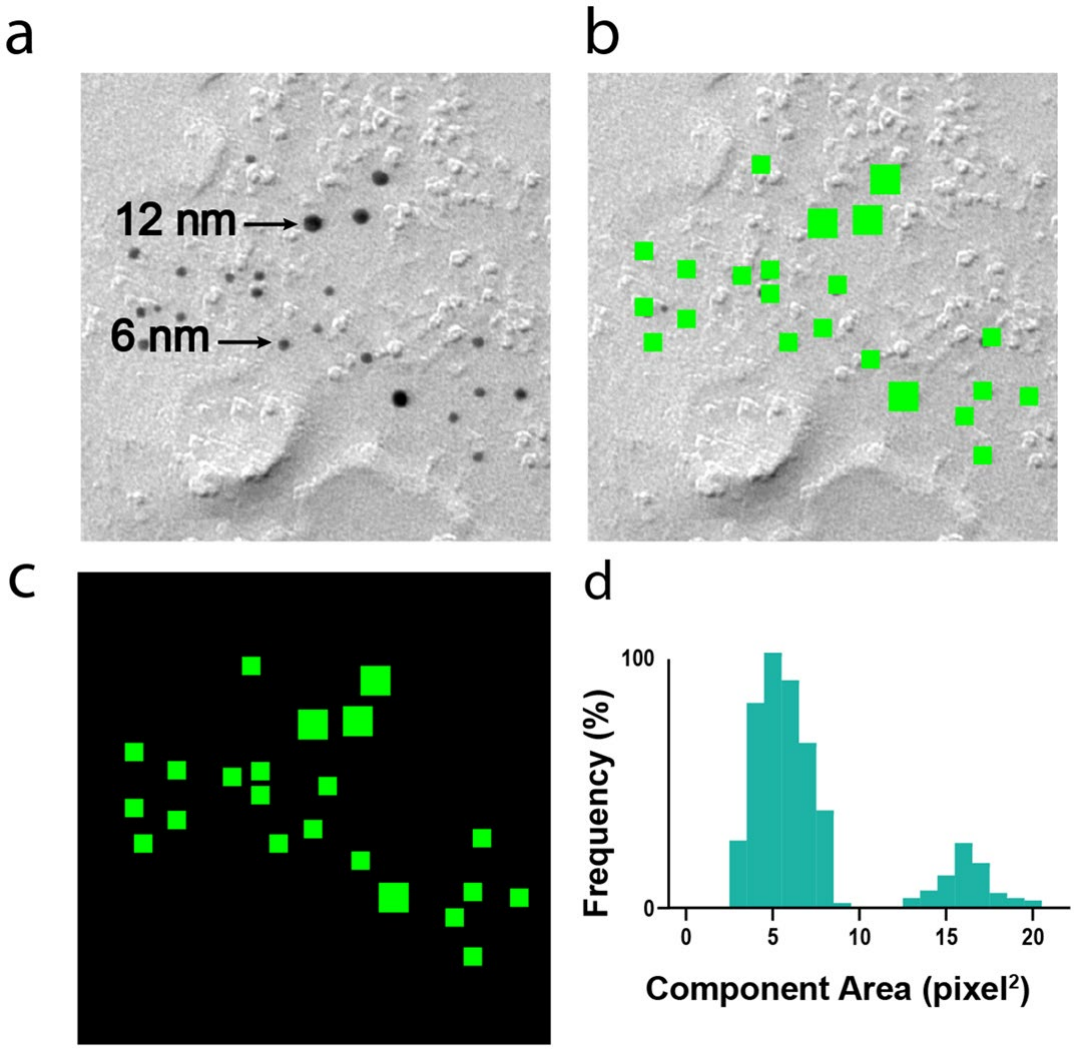
Annotation of individual gold beads. (**a**) Sample input FRIL image having two sizes of gold particles. (**b**) network-annotated output of (**a**). (**c**) Sample network output with background removed. (**d**) Clustering analysis used to group gold particles into two size groups. FRIL images were collected from cerebellar tissue expressing Td-tomato-tagged channelrhodopsin2, and labeled with antibody-conjugated gold nanoparticles.

Generating training data for Gold Digger required identifying colloidal gold particles, overlaying them with a colored mask, and removing the background (Fig. 2a–c). Gold Digger was trained on 6 nm and 12 nm particles. The training dataset was balanced to include images which contained both particle sizes to avoid labeling bias by the algorithm. Thus, in our implementation, Gold Digger creates a mask proportional to the size of the gold particle (Fig. 2b). By building in this functionality, we could increase the utility of the algorithm to enable automated analysis of tissue with multiple immunogold labels, such as when different protein distributions need to be assessed in the same sample using different sized gold particles for each target of interest. We achieved this using connected component analysis, where neighboring pixels in a mask are connected to form object components. By finding the center of mass and surface area of every identified component, Gold Digger can discern clusters from the list of component surface areas in an analyzed image (Fig. 2c). This allows Gold Digger to assign annotations to size groups among the population of identified gold particles (Fig. 2d). Once Gold Digger has assigned gold particles to a group size, it creates a file with the pixel coordinates for every gold particle within that group.

**Figure 3:**
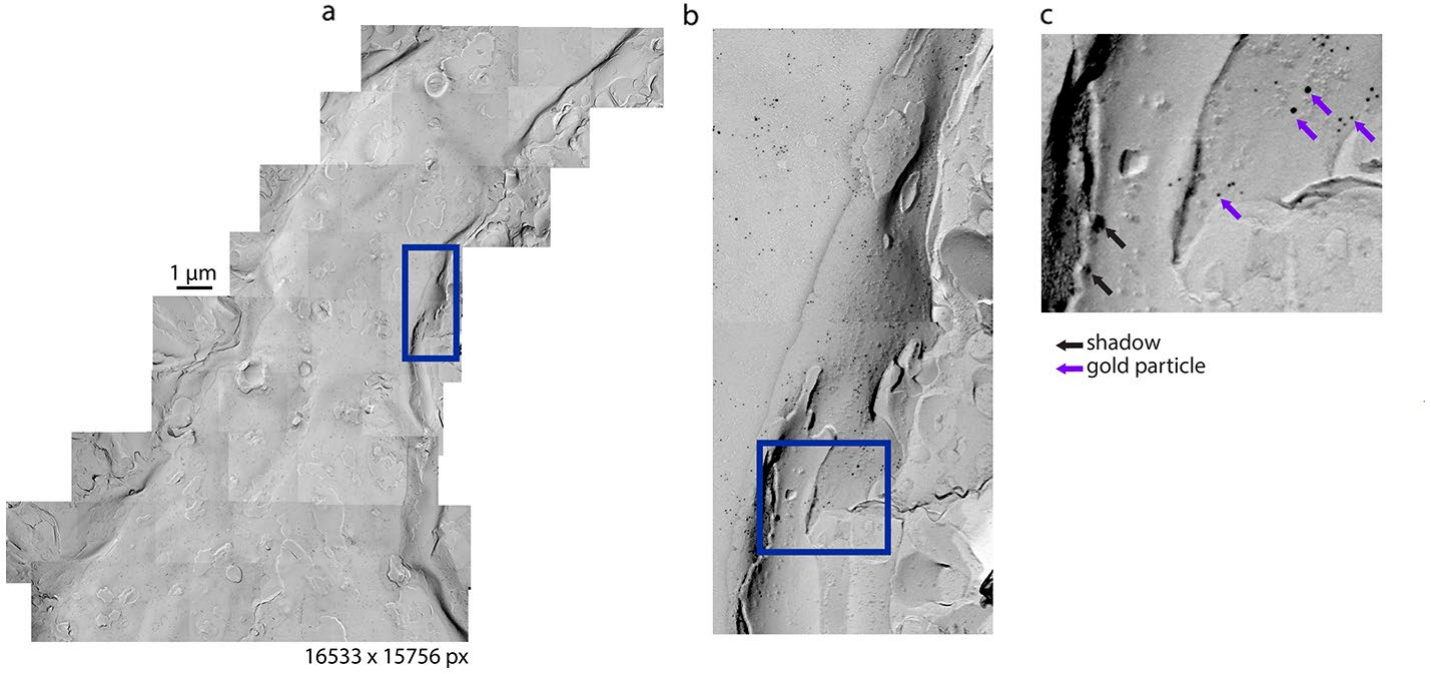
Difficulties of annotating gold particles in FRIL images (**a**) Sample FRIL profile obtained from a Purkinje cell dendrite. Blue box represents area in (**b**). (**b**) Sample area with membrane contours and shadows to illustrate difficulties for thresholding techniques. (**c**) Shadows and gold particles are often difficult to discern with their gray scale. FRIL images were collected from cerebellar tissue expressing Td-tomato-tagged channelrhodopsin2, and labeled with antibody-conjugated gold nanoparticles.

The cGAN was trained to identify colloidal gold particles in FRIL images. As colloidal gold is regular in shape, we reasoned that its detection via an algorithm was feasible. We purposefully attempted to train the network with a maximally heterogeneous representation of FRIL images to avoid overfitting^11^. FRIL datasets are especially large and have characteristically varied background features (Fig. 3a), thus, gold particles in our FRIL training images were populated in the varied backgrounds of our samples that included uneven gray scale and contrast (Fig. 3b) that shifted with the topological structures in the sample that cast local shadows (Fig. 3c). Notably, these features typically present significant challenges to computer-vision-guided gold particle detection. Our final annotated dataset consisted of six different FRIL images obtained from brain tissue that were automatically divided into 3000 sub-sections, each sub-section 256 by 256 pixels as shown in Fig. 1a. By building image sub-sectioning into our analysis workflow, we ensured that our algorithm was able to analyze datasets of any starting size, including those obtained with high-magnification tile scans, which is a common approach for imaging large regions of brain tissue at a resolution necessary for visualizing the colloidal gold particles and for quantifying the fine structure of the long, complex dendritic appendages of neurons (Fig. 3a).

### Network performance

**Figure 4:**
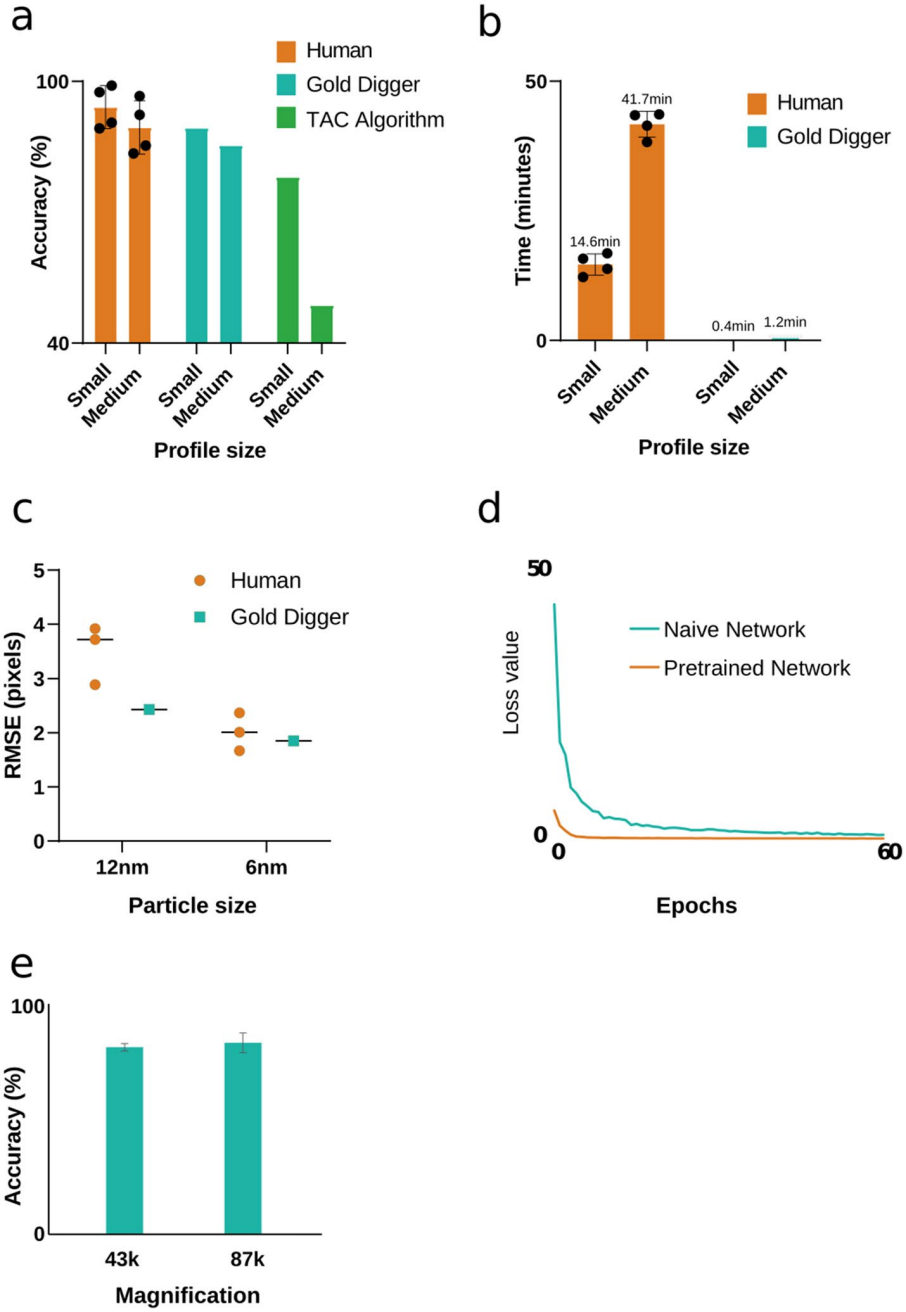
Gold digger performance. (**a**) Gold Digger accuracy vs human and TAC algorithm. Accuracy is calculated by true positives annotated within the p-face of the profile size divided by the total number of gold particles as determined by the ground truth. 100% accuracy was defined by expert annotation. Annotated profiles were collected from trained individuals (n = 4). (**b**) Time required for annotation. Time for manual annotation was gathered by 4 individuals who all had prior immunogold labelling experience. Gold Digger was applied to the dataset locally on a machine equipped with a NVidia GeForce GTX 1080ti. Different computer specifications may lengthen or shorten this time. (**c**) Annotation variability, represented as root mean square error, in Gold Digger compared to humans. (**d**) The pretrained network was trained on the UTZappos-50 k dataset for 200 epochs. (**e**) Gold Digger accuracy relative to original imaging magnification, n = 4.

We compared the ability of Gold Digger to annotate gold particles in EM images to human manual performance. We quantified the time, accuracy, and variability of four different individuals who were asked to annotate gold particles in FRIL images and compared these measurements to those collected from Gold Digger. Two reference images were used (2537 by 3413 pixels; small, and 3326 by 7043 pixels; medium); both images included two different sized gold particles (6 and 12 nm) totaling 612 and 1810 particles, respectively. Gold particle annotations were evaluated by an expert to determine a ground truth for the number of particles, as well as their respective locations. When comparing Gold Digger’s gold-particle annotation accuracy to that of the human annotators, the network performed at a comparable level, independent of gold particle size (Fig. 4a). Gold Digger’s analysis speed, on the other hand, was magnitudes faster than human annotation (Fig. 4b). It is important to note that these reference images were relatively small; tile-scanned FRIL images from brain tissue can easily exceed 16,000 by 1600 pixels (Fig. 3a), enforcing the idea that manually counting gold particles is tedious and slow. Finally, the center-mark locations for gold particles identified by Gold Digger were less variable than those tagged by human annotators, with less variability for larger sized gold particles (Fig. 4c).

To compare Gold Digger’s accuracy to an alternative automated approach, we developed a computer-vision solution based on a previously published algorithm^3^. Briefly, this algorithm applied a fixed parameter gray-level threshold to the image. Next, the remaining binary components were filtered between a predetermined minimum and maximum area parameter. These parameters were chosen based on the known sizes of gold particles contained in the image (i.e., 6 and 12 nm). Lastly, a circularity threshold was applied to each component to validate the common roundness of colloidal gold particles. This threshold-area-circularity (TAC) algorithm showed a high accuracy on the smaller reference image comparable to the level achieved by Gold Digger, though the performance of the TAC algorithm degraded when analyzing the larger reference image, missing nearly half of the gold particles (Fig. 4a). The drop off in performance of the TAC algorithm is likely attributable to the difficulty in establishing an effective, uniform color threshold to differentiate all gold particles in large FRIL images due to the increasing variability of background gray-values inherent in larger, complex images.

### Pretraining and transfer learning

To reduce the amount of training data that was necessary to achieve acceptable gold-particle annotation performance, we pretrained our network using the UT Zappos50k dataset^12,13^. As opposed to training a network from scratch, pretraining is advantageous because it allows the network to use what it learns from one dataset and apply it to the next dataset in a process called transfer learning^14^. Transfer learning allows the weights of the network to update from their random initialization values to ones closer to the weights needed to solve the problem at hand. Although these initial pretraining images pose little resemblance to EM data, this first round of pretraining allows the network to better equip itself for general image analysis by learning the various features which exist in images, such as edges, shades, and shapes. Thus, the cGAN network can discriminate between features in newly introduced training sets more easily, resulting in a decreased need for additional training data.

To gauge the benefit of pretraining Gold Digger, we measured the L1 distance of our generator network, which is also implemented as the loss function used to measure the success of the network during training. We compared the loss value of a naïve network, which saw no prior data, and a network pretrained on the UT Zappos50k dataset. We observed large decreases in both the initial loss value of the pretrained network, as well as the number of training epochs that it took for the loss to converge (Fig. 4d). This was likely the result of the previously mentioned initialization process, which gives pretrained networks a set of learned image processing features. In the same way that it improved the current level of Gold Digger’s performance, pretraining is expected to facilitate future training of the network as well.

### Generalization

Gold Digger uses a deep learning architecture to employ the power of generalization to vastly increase its applicability to heterogeneous data. Generalization is similar to transfer learning obtained through training, as it allows the learned evaluative features of a convolutional neural network to accomplish a similar level of performance on previously unseen data even if that differs significantly from the original training data^10^. We tested Gold Digger’s generalization ability by evaluating its annotation performance on images which were collected based on magnification and sample preparations not present in the training set. While FRIL training images were collected at a 43 k magnification, we employed image scaling to create “faux” magnifications in the training set. We then examined Gold Digger’s ability to identify colloidal gold particles on datasets collected at two different microscope magnifications—43 k and 83 k. Gold Digger quantified gold particles within the same area of interest imaged at both magnifications, achieving a similar accuracy at both resolutions despite its lack of previous training with the high-magnification images (Fig. 4e).

**Figure 5:**
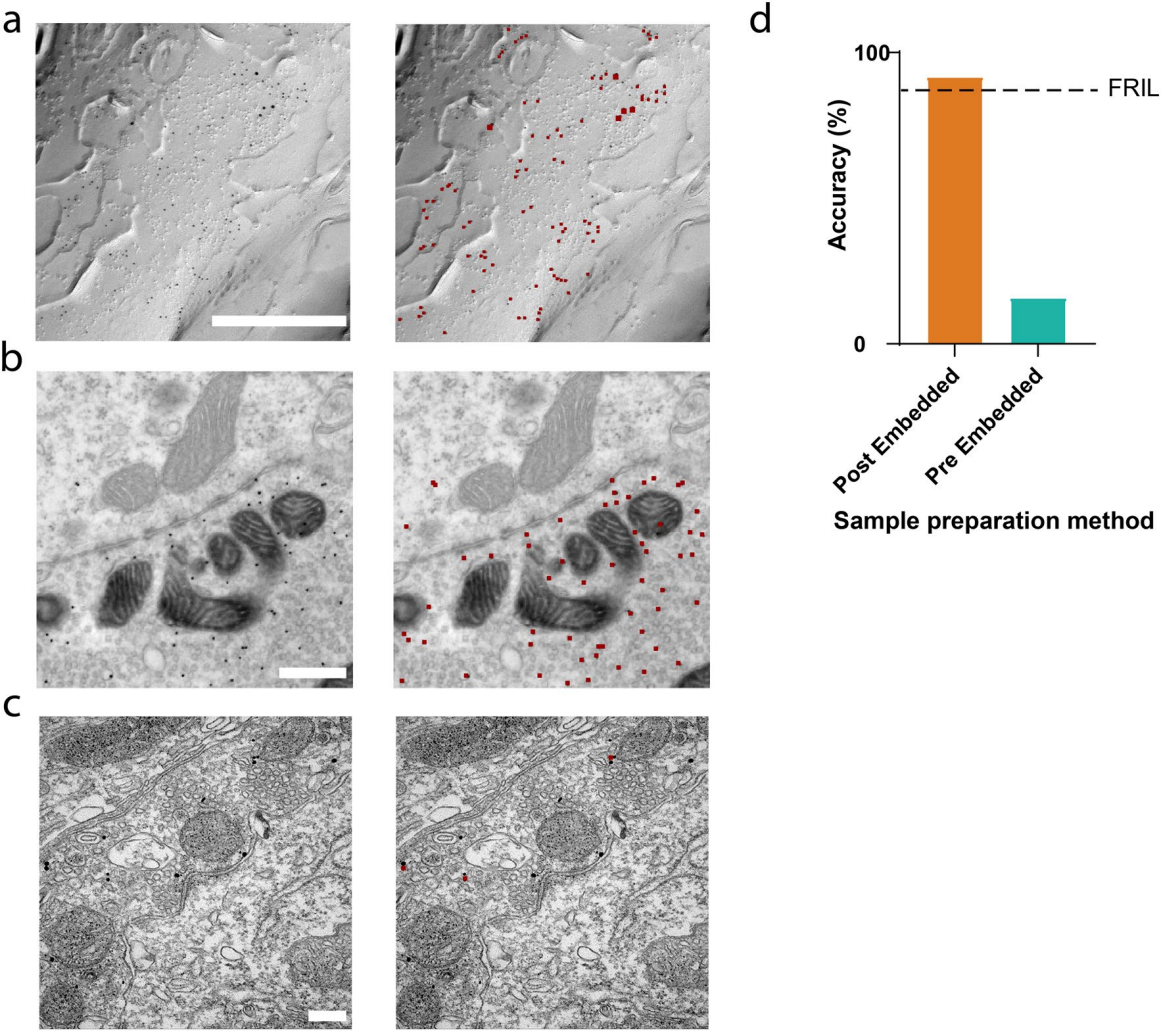
Generalizing to other EM techniques. Left: Sample input images. Right: Sample Gold Digger output – dark red indicates annotated gold beads. (**a**) FRIL sample. Scale bar = 500 nm. (**b**) Post-embedding method sample. Scale bar = 500 nm. (**c**) Pre-embedding method sample scale bar = 200 nm. (**d**) Comparison of labeling accuracy to FRIL method. FRIL images were collected from cerebellar tissue expressing Td-tomato-tagged channelrhodopsin2, and labeled with antibody-conjugated gold nanoparticles. Post-embedded samples = 4, pre-embedded samples = 4.

Colloidal gold particles are used as electron dense markers for labeling FRIL samples as well as those prepared using the post-embedding approach. To determine if Gold Digger could generalize annotation of colloidal gold particles to images obtained from an EM preparation that differed from that used for training, we analyzed data obtained from pre- and post-embedding immunogold labeled brain samples, where the immuno-reaction is performed prior to, and after, embedding the sample into resin, respectively. Pre- and post-embedded sections were immunolabeled with a single size of gold particle (12 nm) and imaged at 43 k magnification (1.11 nm/pixel resolution) (Fig. 5b,c), and compared to results collected using FLIM (Fig. 5a). Although Gold Digger had been trained to identify gold particles only in FRIL images (Fig. 5a), it was able to achieve an accuracy of 91% on post-embedded samples (Fig. 5b,d). However, Gold Digger did much more poorly on pre-embedded samples, achieving an accuracy of 15% (Fig. 5c,d). Overall, these results indicate that Gold Digger does not seem to be narrowly overfit to its training dataset and is capable of annotating colloidal gold particles in images of varying magnifications and sample preparations.

## Discussion

In this report, we present our Gold Digger software for annotating colloidal gold particles in EM images. Gold Digger achieved near human-level accuracy in identifying colloidal gold particles in disparate EM sample preparations. Gold Digger outperformed thresholding-based computer vision techniques^15^ and did not require parameter optimization each time an image changes in quality or contrast. For our needs, we felt that the elimination of adjustable parameters and manual inputs was a particularly high priority, as it slows down the throughput of data analysis and can add additional bias to the results.

Using a deep learning cGAN, we lowered the amount of human input needed to properly annotate gold particles and provide a software tool with an adaptive algorithm for similar annotation tasks. Others may use Gold Digger in its current state or retrain it on different data sets to expand its usability. Thus, Gold Digger can be applied to effectively annotate immunogold on datasets that may be drastically different from those described in this paper. We anticipate that further training will increase accuracy, possibly even surpassing human scores, although this remains to be shown.

Our datasets consisted of only gold particles that had sizes of 6 nm and 12 nm, however upon some inspection, the problem of generalizing magnification is like that of generalizing gold particle size. In 43 k, a 12 nm gold particle may appear as large, in pixel area, as a 6 nm gold particle at a different magnification. Thus, Gold Digger’s performance at different magnifications suggests its ability to also generalize to novel gold particle sizes. Conversely, while changes in magnification are consistent across an image, changes in gold particle size may cause the network to function unexpectedly as the ratio between the particle and the features of the FRIL landscape has changed. Overall, generalization to novel particle sizes requires further testing.

We also found Gold Digger to sometimes struggle with gold particles which are out of focus, as seen in Fig. 5. While Gold Digger does often identify these particles, especially when the model is trained on a heterogeneous dataset including particles that are slightly out of focus, it has trouble identifying their relative size, especially when a large image is composed of a montage of in-focus and out-of-focus parts. Thus, for optimal performance in identifying gold particle sizes, we recommend that images have as homogenous level of focus as possible.

Gold Digger can perform its analysis on an image of any size due the architecture of the network, which means the entire image does not have to be loaded into memory at once. During our testing, we used Gold Digger to analyze images of sizes up to 27,000 by 16,000 pixels, which was the maximum size accommodated by our image handling software (ImageJ/FIJI). Compared to a previously reported method^5^, this is an important advantage when analyzing large images composed of numerous stitched images in tile-scanned datasets. Classic computer vision techniques tend to perform poorly with larger sized images, and their parameters need to be monitored and re-adjusted to adapt to changes in image quality, contrast, etc.^15^. Unlike these techniques, Gold Digger does not lose accuracy when analyzing large images.

In its current state, Gold Digger accepts a single input image for labeling, and returns a comma-separated value (CSV) file containing the pixel coordinate position and pixel area of every identified gold particle. Independent files can be created for different sized gold particles after categorizing all the pixel areas into size-groups using a sorting algorithm. The CSV files can then be used for overlaying the coordinates onto the original image. We used a freely available image processing software (ImageJ/FIJI) to generate overlay images using the original EM image along with the pixel coordinates from the CSV file. This graphical view makes manual corrections of mislabeled gold particles by users more approachable. This CSV file can also be easily written into a preferred computing language and used for further analysis, such as nearest-neighbor distance statistics or cluster analysis of the particles. All code needed to run Gold Digger as well as any external scripts are open source, and can be found at the github link listed in the methods sections of this report.

## Methods

### Animals

All animal procedures used in this study were approved by the Max Planck Florida Institute for Neuroscience Animal Care and Use Committee. All procedures were performed in compliance with the ARRIVE guidelines.

### Sample preparation and imaging for FRIL

In this study, we used 8-to-12-week-old mice. Experimental animals were obtained by crossing the Pcp2-Cre driver line with a reporter line (Ai27) to yield membrane expressed Td-tomato-tagged channelrhodopsin2 specifically in cerebellar Purkinje cells^16,17^. For sample preparation, mice were anesthetized with a mixture of ketamine/xylazine and were then perfused transcardially with 0.9% NaCl followed by 4% paraformaldehyde in 0.1 M Sorensen’s phosphate buffer (PB). The cerebellum was isolated by dissection and 150 μm-thick sagittal slices were sectioned using a vibratome (Leica VT1200). Slices containing cerebellar lobules Crus I and Crus II lobes were serially incubated in 10%, 20% (for 30 min each), and then 30% glycerol (at 4 °C overnight) for cryoprotection. A biopsy punch was used to isolate a small sections of tissue (1.2 mm in diameter) that were then frozen using a high-pressure freezing machine (HPM100, Leica). FRIL was performed as described previously^18^. Briefly, the frozen samples were fractured into halves using a double replication device at − 140 °C. They were replicated with layers of 2–4 nm carbon followed by 2 nm carbon-platinum and then 30–40 nm carbon using a JFDII freeze fracture machine (JEOL/Boeckeler). The tissue was dissolved in a digesting solution containing 2.5% sodium dodecyl sulfate (SDS), 20% sucrose and 15 mM Tris–HCl (pH 8.3) with gentle agitation (82.5 °C for 18 h). After digestion, the replicas were washed and blocked with 4% bovine serum albumin (BSA) and 1% cold fish skin gelatin (FSG) in 50 mM Tris buffered saline (1 h). The replicas were then incubated with a mixture of primary antibodies including rabbit anti-red fluorescent protein (4 µg/ml, Rockland cat# 600–401-379) and mouse anti-Kv4.3 K75/18.1 IgG1 (40 µg/ml Neuromab) diluted in 4% BSA and 1% FSG in TBS (18 h at room temperature). The replicas were then incubated in a mixture of secondary antibodies including donkey anti-rabbit IgG conjugated to 6 nm gold particles at 1:30 dilution (Jackson ImmunoResearch 711-195-152) and donkey anti-mouse IgG conjugated to 12 nm gold particles at 1:30 dilution (Jackson ImmunoResearch 715-205-150), diluted in 4% BSA and 1% FSG in TBS (18 h at room temperature). After washing, the replicas were placed on copper aperture grids and examined with a Tecnai G2 Spirit BioTwin transmission electron microscope (Thermo Fisher Scientific) at 100 kV acceleration voltage. Images were taken with a Veleta CCD camera (Olympus) controlled by a TIA software (Thermo Fisher Scientific). To obtain sufficient resolution for gold particle identification, we used 43,000 × magnification as the microscopy indication, which gave 1.11 nm/pixel for the 2000 × 2000 pixels digital image. Some samples were also re-imaged at 87,000 × magnification (0.55 nm/pixel resolution). To image the soma and dendrites of the same Purkinje cells at high resolution, we used tile-imaging. The tiles were stitched and the brightness and contrast of images were adjusted in Photoshop (Adobe CS6), then merged and saved as TIFF files that could then be used by the Gold Digger software.

### Sample preparation and imaging for post-embedding immunogold labeling

Sample preparation for Immuno EM was done in accordance with established protocols^19^. Briefly, P7 or P21 mice were anesthetized with an intraperitoneal injection of tribromoethanol (250 mg/kg of body weight, and perfused transcardially with 0.9% NaCl in MilliQ water followed by a fixative containing 2% paraformaldehyde and 1–2% glutaraldehyde in 0.1 m cacodylate buffer (CB), pH 7.4, containing 2 mm CaCl_2_. Brains were extracted and postfixed in the same fixative for 2 h, then washed in CB. The brainstem was sliced coronally at a thickness of 50 μm (TEM) using a Leica VT 1200S vibratome in 0.1 m CB. The EGFP expression at the injection site and in the contralateral MNTB (medial nucleus of the trapezoid body) was confirmed under an epifluorescence microscope (CKX41, Olympus). All EM images of calyx terminals were taken from the middle region of the MNTB.

Sections of 100 nm thickness were obtained using a Leica UC7 ultra-microtome, collected onto a 100-mesh nickel grid and air-dried. As an antigen unmasking procedure for Durcupan resin, grids were placed section side down on water heated on a hotplate to 93–95 °C for 10 min^20^. Following heat treatment, the water was slowly cooled down for 15 min by mixing in room temperature water. Without drying, grids were immediately placed onto drops of blocking medium for 10 min composed of 0.1% Tween 20, 1% bovine serum albumin, 1% normal goat serum, 1% fish skin gelatin, and 0.005% sodium azide diluted in Tris-buffered saline (TBS) buffer, pH 7.4. Grids were then incubated on a chicken anti-GFP primary antibody solution (0.5 mg/ml; Abcam, catalog #ab13970) diluted 1:100 in the same blocking medium, and placed in a humid chamber overnight at room temperature. They were then washed and secondary antibody incubation was performed for 1 h at room temperature with a 12 nm colloidal gold donkey anti-chicken antibody (Jackson ImmunoResearch, catalog #703-205-155) diluted at 1:30 in blocking medium. Grids were washed again on TBS and the reaction was stabilized by placing them on 1% glutaraldehyde in PBS for 5 min. Grids were rinsed on water and counterstained with uranyl acetate and lead citrate, and examined in a Tecnai G2 Spirit BioTwin transmission electron microscope (ThermoFisher Scientific) at 80 kV acceleration voltage. Images were taken with a Veleta CCD camera (Olympus) operated by TIA software.

### Network construction and training

Gold digger is built as a wrapper on pix2pix cgan^21,22^. It runs using Python 3.7 and the PyTorch machine learning library. The image generating-network is a Unet-256 (with 256 layers), trained with a batch size of 2, over 200 epochs, with a linear learning policy and learning rate of 2e−4. All relevant code is available on github. For training Gold Digger, we used six different montaged FRIL images, stitched together from between 2 and 60 of 2,048 × 2,048 pixel digital images obtained at 43,000 × magnification (1.11 nm/pixel). The montaged images were manually annotated by trained EM specialists to identify and annotate all gold particles. The montaged images were automatically sub-sectioned into 256 by 256-pixel subsections, resulting in 4500 analysis sections in total. Subsections devoid of gold particles and were removed to avoid inclusion in the training dataset resulting in a total of approximately 3000 subsections. To eliminate training bias, we balanced our training data so that there was an equal number of inputs with and without gold particles. Image sections were processed by the cGAN for training over 200 on a Nvidia GTX 1080ti graphical processing unit lasting a total of 22 h.

## Data Availability

All code for Gold Digger is available via github at https://github.com/mpfi-dsp/GD_Terminal. TAC Algorithm: https://github.com/mpfi-dsp/TACAlgorithm. The data presented in this study is available to interested parties by contacting the corresponding author.
