# Author Correction: A deep learning approach to identifying immunogold particles in electron microscopy images

**DOI:** 10.1038/s41598-021-00540-y

**Published:** 2021-10-22

**Authors:** Diego Jerez, Eleanor Stuart, Kylie Schmitt, Debbie Guerrero-Given, Jason M. Christie, William E. Hahn, Naomi Kamasawa, Michael S. Smirnov

**Affiliations:** 1 Max Planck Florida Institute for Neuroscience, 1 Max Planck Way, Jupiter, FL 33458 USA; 2 Department of Mathematical Sciences, Florida Atlantic University, 777 Glades Rd, Boca Raton, FL 33431 USA

Correction to: *Scientific Reports* 10.1038/s41598-021-87015-2, published online 08 April 2021

William E. Hahn was omitted from the author list in the original version of this Article.

The Author Contributions section now reads:

“Conceptualization: D.J., J.M.C., N.K., W.E.H., and M.S.; Investigation: D.J., E.S., K.S., and D.G-G.; Writing and Editing, D.J., E.S., D.G-G., J.M.C., N.K., W.E.H., and M.S.; Funding Acquisition: J.M.C.; Supervision: J.M.C, N.K., W.E.H., and M.S.”

In addition, Diego Jerez and Eleanor Stuart were incorrectly affiliated with ‘Florida Atlantic University, 777 Glades Rd, Boca Raton, FL, 33431, USA’. Their correct affiliation is listed below.

Max Planck Florida Institute for Neuroscience, 1 Max Planck Way, Jupiter, FL, 33458, USA.

Finally, an incorrect email address for author Michael S. Smirnov was quoted. Correspondence and requests for materials should be addressed to msmirnovs@gmail.com.

The original Article has been corrected.

